# Editorial: Inter-organ communication beyond mammals: the role of tissue-specific cytokines

**DOI:** 10.3389/fendo.2023.1208000

**Published:** 2023-05-19

**Authors:** Emilio J. Vélez, Encarnación Capilla, Esmail Lutfi

**Affiliations:** ^1^ Université de Pau et des Pays de l’Adour, E2S UPPA, Institut national de recherche pour l’agriculture, l’alimentation et l’environnement (INRAE), UMR1419 Nutrition Métabolisme et Aquaculture, Saint-Pée-sur-Nivelle, France; ^2^ Department of Cell Biology, Physiology and Immunology, University of Barcelona, Barcelona, Spain; ^3^ Nutrition and Feed Technology, NOFIMA (Norwegian Institute of Food, Fisheries and Aquaculture Research), Ås, Norway

**Keywords:** adipokines, myokines, osteokines, tissues crosstalk, interleukins (IL), growth hormone, IGFs

Each tissue type composing an organism has unique characteristics related to the functional organ it belongs to, and this is reflected in its cellular metabolism and development. However, the tissues are not isolated units but rather communicate with each other through signaling molecules called cytokines. Cytokines help the organism perceive both external and internal cues at a more holistic level, allowing for integrative physiological responses that can adapt the organism to changing situations. This Research Topic compiled studies on the effects of cytokines and the impact of their inter-tissue crosstalk on whole-body homeostasis in different physiological situations in both mammals and fish, including model organisms and farmed species. The topic includes seven contributions in total, four original research articles, two reviews, and one minireview, covering different topics ranging from endocrine regulation of the circadian rhythm and the role of interleukins (ILs) in human diseases to the most recent advances on inter-tissue crosstalk in fish species.

Senesi et al. reviewed how circadian rhythms are orchestrated in response to different stimuli through a complex neuronal and endocrinological network system. The circadian rhythm regulates several hormones, transcription factors, and enzymes associated with the control of food intake (1) and modulates cardiac parameters fluctuation. In this sense, disturbances in diet and eating behaviors are associated with rhythm desynchronizations and the potential onset of metabolic diseases such as obesity. Accordingly, circadian rhythm misalignments are considered a risk factor for cardiometabolic pathologies. The authors highlighted the potential of a designed meal plan (considering composition and timing) to re-synchronize the circadian rhythm, positively influencing the cardiometabolic status and patients’ health. Human health is a global concern, especially so since the coronavirus disease (COVID-19) outbreak. The severe acute respiratory syndrome coronavirus 2 (SARS-CoV2) infection is associated with excessive production of cytokines, including some ILs (e.g., IL-1β or IL-6), which induces hyper-immune activation and promotes systemic inflammation, increasing death risk. Alves et al. discussed the anti-inflammatory effects of the adipo-myokine IRISIN, which is normally produced after exercise (2), and how increased levels of IRISIN could prevent excessive inflammation accompanying COVID-19 and reduce severe outcomes of the disease. Inflammation also plays a role in the development of the autoimmune ocular disease known as thyroid-associated ophthalmopathy (TAO) (3). Wu et al. explored the role that IL-11 plays in the orbital fibrosis associated with TAO. The authors reported increased levels of IL-11 in TAO patients that positively correlated with their clinical activity score and highlighted its potential as a therapeutic target candidate for the treatment of TAO. Furthermore, while some ILs are involved in the course of COVID-19 and TAO diseases, others, such as IL-10, play an anti-inflammatory role (4). Subramanian et al. evaluated the levels of IL-10 in both non-obese and obese individuals, differentiating between healthy subjects and patients with type 2 diabetes and considering potential gender differences. The authors found that type 2 diabetes alone does not regulate IL-10 and suggested that IL-10 could exert a protective effect on the chronic inflammation associated with obesity in women.

Inter-organ communication between tissues is relatively well recognized in mammals (5); however, it remains unknown whether it also occurs in other groups of vertebrates including fish. Hue et al. reviewed the state-of-the-art of the local effects of myokines, adipokines, and osteokines in fish, and then discussed their inter-tissue effects. The authors provided evidence of the existence of inter-tissue communication beyond mammalian species and pointed out new and interesting approaches, including *in vitro* tools, which will increase our understanding of tissue crosstalk in this group of vertebrates. In this regard, Otero-Tarrazon et al. characterized the local expression of several cytokines and tissue-specific regulatory factors in a very particular situation, as in the case of skeletal muscle regeneration after mechanical damage, in both muscle and vertebral bone samples from injured and control gilthead sea bream fish. The results showed early upregulation of the cytokines IL-6 and IL-15 after injury, followed by either upregulation or downregulation in the bone of some genes such as *myostatin*, whose inter-tissue inhibitory role was previously reported in mammals (6). Lastly, Link et al. investigated the orchestration of cytokines and growth factors in different tissues in blackchin tilapia, a fish species that naturally faces large variations in salinity (7), during the physiological adaptation of fish to changing salinities. The results provided new information on the role of the growth hormone/insulin-like growth factors (GH/IGFs) axis in such a process.

Taken together, the compiled works within this Research Topic highlighted the importance of considering both inter-organ and inter-tissue communications to better understand health and disease, and emphasized that this crosstalk is not restricted to mammals, as it undoubtedly also occurs in fish.

## Author contributions

EV, EC, and EL drafted and critically reviewed the manuscript. All authors contributed to the article and approved the submitted version.
